# Qualitative and Quantitative Effects of Fatty Acids Involved in Heart Diseases

**DOI:** 10.3390/metabo12030210

**Published:** 2022-02-25

**Authors:** Hidenori Moriyama, Jin Endo, Hidehiko Ikura, Hiroki Kitakata, Mizuki Momoi, Yoshiki Shinya, Seien Ko, Genki Ichihara, Takahiro Hiraide, Kohsuke Shirakawa, Atsushi Anzai, Yoshinori Katsumata, Motoaki Sano

**Affiliations:** Department of Cardiology, Keio University School of Medicine, 35 Shinanomachi, Shinjuku-City, Tokyo 160-8582, Japan; h.moriyama@keio.jp (H.M.); ikurahidehiko@keio.jp (H.I.); kitkatmed@yahoo.co.jp (H.K.); m.momoi@keio.jp (M.M.); midnight.1119@gmail.com (Y.S.); kao227@hotmail.com (S.K.); genki@z5.keio.jp (G.I.); hiraiday@hotmail.com (T.H.); shirakawa19840905@gmail.com (K.S.); atsushi_anzai@hotmail.com (A.A.); goodcentury21@gmail.com (Y.K.); msano@keio.jp (M.S.)

**Keywords:** fatty acid, heart failure, lipid dynamics, lipotoxicity, lipid droplet, cardiolipin, lipid mediator, n-3 PUFA

## Abstract

Fatty acids (FAs) have structural and functional diversity. FAs in the heart are closely associated with cardiac function, and their qualitative or quantitative abnormalities lead to the onset and progression of cardiac disease. FAs are important as an energy substrate for the heart, but when in excess, they exhibit cardio-lipotoxicity that causes cardiac dysfunction or heart failure with preserved ejection fraction. FAs also play a role as part of phospholipids that compose cell membranes, and the changes in mitochondrial phospholipid cardiolipin and the FA composition of plasma membrane phospholipids affect cardiomyocyte survival. In addition, FA metabolites exert a wide variety of bioactivities in the heart as lipid mediators. Recent advances in measurement using mass spectrometry have identified trace amounts of n-3 polyunsaturated fatty acids (PUFAs)-derived bioactive metabolites associated with heart disease. n-3 PUFAs have a variety of cardioprotective effects and have been shown in clinical trials to be effective in cardiovascular diseases, including heart failure. This review outlines the contributions of FAs to cardiac function and pathogenesis of heart diseases from the perspective of three major roles and proposes therapeutic applications and new medical perspectives of FAs represented by n-3 PUFAs.

## 1. Introduction

Many lipids are subjected to precise enzymatic control to maintain the homeostasis of tissues in living organisms, and it is important to regulate the qualitative and quantitative balance of lipids in the heart. In the stressed heart, alterations in lipid composition and structural remodeling of membrane lipids occur mainly through changes in the expression of enzymes related to lipid synthesis, metabolism, remodeling, and oxidation. The changes in the cardiac lipid profile act pathologically or compensatory to heart injury and characterize heart failure.

Fatty acids (FAs) are used as (1) energy sources, (2) components of membrane phospholipids, and (3) bioactive mediators. In this review, from the point of view of these three major roles, we introduce and discuss the recent advances in understanding lipid dynamics, especially focusing on FA changes in heart failure.

## 2. FAs Control the Heart as an Energy Source

**Figure 1:**
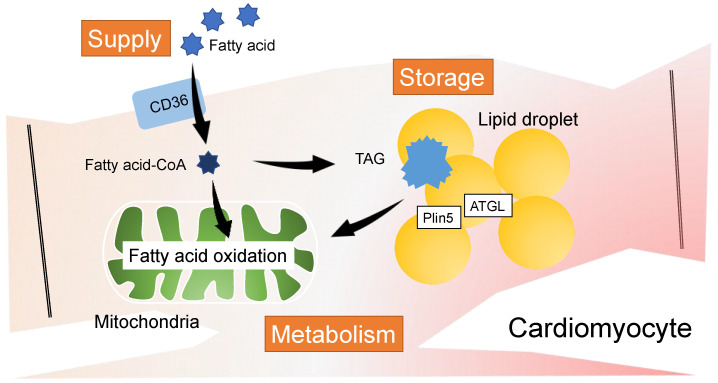
FA dynamics in cardiomyocytes. The supply, storage, and metabolism of the FAs are strictly controlled in healthy hearts. TAG, triacylglycerol; ATGL, adipose triglyceride lipase; Plin5, perilipin 5.

In the heart, FA uptake, storage, and metabolism are strictly regulated to produce adenosine triphosphate (ATP) in the mitochondria via FA oxidation (FAO) (Figure 1). Excessive supply of FAs to the heart due to overeating or metabolic disorders is known to produce excess energy and damage cardiomyocytes by lipotoxicity [1]. In fact, lifestyle diseases such as obesity, diabetes, and metabolic syndrome are closely associated with the pathogenesis of heart disease. In addition, “heart failure with preserved ejection fraction (HFpEF)” due to left ventricular (LV) diastolic dysfunction, which has been increasing in number in recent years, has obesity and diabetes as risk factors, and it has been pointed out that cardiac lipotoxicity is involved in the pathogenesis [2,3]. Additionally, some of the oversupplied FAs are not only consumed but are also stored as lipid droplets (LDs). In addition to functioning as a reservoir, LDs play important roles in cell survival, including the regulation of lipid dynamics and antioxidant stress action. In recent years, the molecular mechanism underlying the myocardial damages caused by abnormal cardiac lipid metabolism resulting from systemic metabolic disorders is gradually being elucidated. This chapter outlines cardiac lipotoxicity and LD multifunctionality due to excessive FA loading.

### 2.1. Cardiac Lipotoxicity Due to the Imbalance between Supply and Oxidation of FAs

Free FAs are taken up into cardiomyocytes via transporters, such as CD36 and FA transport protein (FATP), and are then rapidly converted to acyl-CoA by long-chain acyl-CoA synthase (ACSL) [4]. Acyl-CoA undergoes β-oxidation in mitochondria to produce ATP. Cardiac dysfunction caused by abnormal lipid metabolism in cardiomyocytes is called lipotoxic cardiomyopathy. Lipotoxicity is observed not only on the left heart but also on the right heart failure due to pulmonary hypertension [5]. The molecular mechanism of cardiac lipotoxicity is complex, and the definitive mechanism by which lipid overload leads to cardiac dysfunction and HFpEF remains unclear. Previous reports have presented that lipotoxicity is involved in mitochondrial dysfunction, autophagy disruption, reactive oxygen species (ROS) production, endoplasmic reticulum (ER) stress, and cardiotoxic lipid (such as ceramide or diacylglycerol) accumulation [6]. In obesity and diabetes, the supply of FAs is increased, and FAO is enhanced [7,8]. Increased FAO in the heart has long been thought to lead to cardiac dysfunction due to overproduction of ROS and decreased mitochondrial function. However, recent studies have shown that the upregulation of FAO by removal of the acetyl-coenzyme A carboxylase 2 (ACC2), which inhibits the transport of FA to mitochondria, attenuates cardiac dysfunction caused by metabolic stress from a high-fat diet (HFD) or pressure overload by transverse aortic constriction [9,10]. From the above, it is considered that the imbalance in which the FA supply exceeds the FA oxidation characterizes the lipotoxicity of the heart and that the elimination of this imbalance contributes to the improvement of cardiac dysfunction [4].

Patients with HFpEF account for about half of all patients with heart failure, and recently, the number of patients has been increasing. However, there is little evidence of clinically effective treatments [11,12]. HFpEF is a typical phenotype of lipotoxic cardiomyopathy because of the presence of excessive lipid accumulation in the heart or the presence of major risk factors, such as obesity and metabolic dysfunction [13,14]. To date, HFD- or obesity-induced HFpEF animal models have commonly been used. Recently, a new animal model has been developed that combines mechanical stress (L-arginine methyl ester [L-NAME]) in addition to metabolic stress (HFD) as an HFpEF model that more accurately mimics the pathology [15]. In this novel HFpEF model, the poor activation of the X-box-binding protein-1(XBP1) which is one of the key molecules of unfolded protein response increased the abundance and activation of the transcription factor Forkhead box protein O1 (FoxO1) responsible for lipid metabolism, resulting in excessive lipid accumulation in cardiomyocytes [16]. Interestingly, FAO does not increase in HFpEF [16]. These results also suggest that FAO is not adaptively activated, and abnormal lipid accumulation occurs in cardiomyocytes, which induces cardiotoxicity and contributes to the appearance of HFpEF.

### 2.2. Oversupplied FAs Are Stored in LDs

Some of the oversupplied FAs are stored in the form of triacylglycerols (TAGs) in LDs. LDs have a stable structure with a single layer of phospholipids surrounding the TAGs. Notably, LDs in cardiomyocytes are smaller than those in adipocytes, and the TAGs stored in LDs are enzymatically released in response to energy demand and used for β-oxidation. LDs are spatially located near mitochondria, and the functional and structural interactions between LDs and mitochondria enable an accurate and rapid supply of FAs [17]. Therefore, dysregulation of LDs has been implicated in various cardiovascular diseases, including heart failure [18].

In cardiomyocytes, the TAG hydrolysis enzyme, adipose triglyceride lipase (ATGL), and the scaffold protein, perilipin 5, are highly expressed on the surface of LDs, and play an important role in the turnover of TAG in myocardial LDs [19,20]. Since ATGL is the rate-limiting enzyme for TAG breakdown in lipolysis, ATGL-deficient mice develop heart failure early and die due to decreased lipolysis and excessive accumulation of TAG in the heart [21]. The ATGL gene also causes the novel and intractable disease triglyceride deposit cardiomyovasculopathy, which progresses to advanced heart failure [22]. Interestingly, the hearts of ATGL-deficient mice exhibit reduced expression of target genes for peroxisome proliferator activated receptor alpha (PPARα) and oxidative phosphorylation in mitochondria [21,23]. The nuclear receptor PPARα is a transcription factor that comprehensively controls FA uptake, TAG synthesis, and oxidation. A decrease in PPARα activity, and not an excessive accumulation of TAG, leads to cardiotoxicity in ATGL-deficient mice.

In addition to excess lipids, changes in the quality of lipids also affect the properties and functions of the heart. FA desaturation has many beneficial roles in cardiomyocytes, such as increasing the fluidity of cell membranes and suppressing ER stress, but desaturated FAs are still susceptible to oxidation. Lipid oxidation often has detrimental effects on human health. Since low-density lipoprotein (LDL) contains PUFAs such as linoleic acid, it is easily oxidized by ROS and radicals. Uptake of oxidized LDL by macrophages generated foam cells that accumulated LDs, leading chronic inflammation and smooth muscle proliferation, resulting in advanced atherosclerosis [24]. Taking advantage of the oxidizable properties of PUFAs, LDs also have a role as absorbers to take on lipid oxidative damage to cells. In the neural stem cell niche exposed to hypoxia and oxidative stress, polyunsaturated FAs (PUFAs) move from the cell membrane to the LDs, preventing cellular peroxidation [25].

The composition of LDs depends on the metabolic environment of the tissue in which LDs are accumulated. LDs in cardiomyocytes mainly store TAG, while steroid-producing cells such as macrophages and adrenocortical cells mainly store CE [26]. Although one study reports on the differences in the proteome expressed in triglyceride-rich LDs and cholesterol-rich LDs [27], the role of cholesterol-rich LDs in cardiomyocytes and its relationship to heart disease remain unclear.

## 3. FAs Control the Heart as a Component of Membrane Phospholipids

FAs play an important role as major components of phospholipids. Many studies have shown that dysregulation and disruption of lipid dynamics in cell and mitochondrial membranes are associated with heart failure. Importantly, FAs not only form bilayer membranes as part of phospholipids but also function as lipid metabolites with physiological activity by undergoing various oxidative and metabolic processes in the cell membranes (see Section 4). The diversity of FA profiles in phospholipids greatly affects cell function through changes in cell membrane fluidity and the production of bioactive lipids. Glycerophospholipids have different properties due to the binding of choline, ethanolamine, serine, or inositol to the phosphate group, but the composition of FA esters bound to the glycerol backbone also greatly affects the functional diversity of phospholipids [28]. Generally, saturated FAs (SFAs) and monounsaturated FAs are ester-bonded to the sn-1 position, and PUFAs are bonded to the sn-2 position of the glycerol backbone, respectively. The composition of FAs with different chain lengths and the number of unsaturated bonds are defined by each tissue and cell.

**Figure 2:**
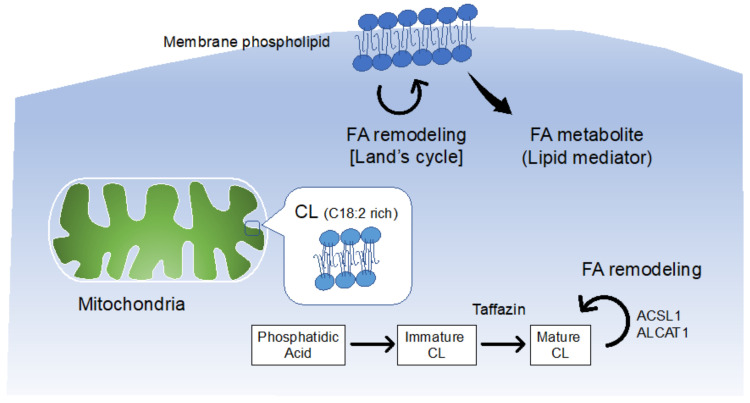
Intracellular dynamics of phospholipid-containing FAs. The diversity of the FAs is characterized by the action of various enzymes involved in the synthesis and remodeling processes. FA, fatty acid; CL, cardiolipin.

Phospholipids are not only produced from glycerol-3-phosphate in the de novo Kennedy pathway [29]; various phospholipases and lysophospholipid acyltransferases are involved in the remodeling process (Lands’ cycle) [30,31], resulting in the structural diversity of phospholipids. In recent years, these enzymes which are involved in lipid remodeling and various pathological conditions have been identified (Figure 2). Here, we outline the cardiac effects of FA composition in phospholipids, including cardiolipin (CL), a phospholipid abundant in the heart.

### 3.1. Balance of FA Saturation in Membrane Phospholipids

Changes in FA composition observed in heart disease are regulated, at least in part, by the FA synthesis system. The most abundant FAs have 16 or 18 carbon molecules, which are major products of endogenous FA synthesis and are essential for cellular activity. Furthermore, the addition of double bonds and the extension of carbon chains are enzymatically conducted to produce unsaturated FAs.

Several reports have shown the differential effects of saturated FA and monounsaturated FA on cellular and cardiac function [32,33], while desaturation enzymes are associated with heart disease. Stearoyl-CoA desaturase 1 (SCD-1) introduces a double bond into C16:0 or C18:0 saturated FAs to produce C16:1 (n-7) or C18:1 (n-9) monounsaturated FAs. SCD-1 is highly expressed in the hearts of patients with obesity or diabetes, and an increase in SCD-1 alleviates SFA-induced adverse FA catabolism and eventually prevents SFA-induced apoptosis [34]. Moreover, SCD-1 is regulated by Sirtuin 1 (*SIRT1*), a well-known longevity gene, and acts suppressively against SFA overload-induced membrane phospholipid saturation to restore LV diastolic dysfunction. Therefore, in one study, the administration of nicotinamide mononucleotide, which enhances the activity of Sirt1, improved the LV diastolic function of SFA-rich HFD-fed mice [33,35].

There is accumulating evidence that the desaturase enzyme family, fatty acid desaturase (FADS), is also associated with cardiovascular diseases. FADS2 (also known as δ-6 desaturase [D6D]) is a rate-limiting enzyme that produces long-chain PUFAs, such as arachidonic acid (AA) from linoleic acid (LA) or docosahexaenoic acid (DHA) from α-LA. Since FADS2 activity is increased in the hearts of patients with dilated cardiomyopathy, LA levels are lower and levels of AA and DHA are reciprocally higher in the hearts of patients compared to the hearts of healthy controls [36]. The alteration of the FA profile is attenuated in heart failure chronically unloaded with an LV assist device. Furthermore, the administration of D6D inhibitors prevents remodeling of FA composition and attenuates myocardial elevations in pathogenic eicosanoid species and lipid peroxidation to suppress cardiac hypertrophy, fibrosis, and contractile dysfunction [36,37], indicating that aberrant activation of FADS2 has a pathogenic role that exacerbates heart failure through unwanted desaturation of FAs.

From the above evidence, the balance between the saturation and unsaturation of FAs in the heart is important for maintaining organ homeostasis. Some desaturase enzymes play a role in protecting against cellular damage due to over-saturation of cardiac FA composition, while others cause over-desaturation to promote lipid peroxidation and the production of inflammatory mediators.

### 3.2. FA Remodeling in Cardiolipin and Heart Disease

CL is a major phospholipid that constitutes the mitochondrial membrane and is essential for maintaining the function of mitochondrial proteins involved in energy production [38,39]. CL is localized to the inner mitochondrial membrane in healthy cells. However, the distribution is dramatically changed upon mitochondrial injury and depolarization, when a significant portion of CLs is translocated to the outer mitochondrial membrane, and they are associated with apoptosis and mitophagy [40,41,42].

CL is a diphosphatidylglycerol with a dimeric structure, and in the mitochondria of healthy human hearts, tetra-linoleic-CL with C18:2 (n-6) four acyl chains (symmetrical CL) comprises approximately 80% of CL [43]. Loss of CL and/or tetra-linoleic-CL results in dysfunction of the oxidative phosphorylation machinery, elevated production of ROS, and alterations of mitochondrial morphology [44]. The amount of CL and its FA composition (mainly C18:2 content) are altered in various pathological human and animal models of heart failure, ischemia/reperfusion injury, and diabetic cardiomyopathy [45,46,47]. These alterations are also observed in both left and right ventricular diseases [48]. The symmetry of CL is lost if it contains at least one different FA residue or oxidative modification, and such an asymmetric CL is involved as a precursor signaling molecule [40]. In addition, when CL is oxidized that electrostatically holds cytochrome C, the cytochrome C is released and accumulates in the inter-membrane space, triggering apoptosis. Thus, oxidized CL is closely related to acute tissue damage, including cardiac ischemia/reperfusion injury [49,50,51]. Furthermore, positively charged doxorubicin, which is a well-known anticancer drug that causes cardiomyopathy, is attracted to the highly negatively charged CL, and the doxorubicin and generated ROS oxidize the CL, causing structural change, cytochrome C release, and disruption of the electron transfer system [52].

CL is first produced in a highly saturated FA-rich form of phosphatidic acid (PA) through a de novo synthetic pathway, and it matures by incorporating unsaturated chains, such as C18:2 via the re-acylation pathway [45]. Proper remodeling and maintenance of C18:2 chain content in CL play important roles in maintaining normal heart functioning.

Variants of the gene encoding the re-acylation enzyme of CL, tafazzin, cause Barth syndrome, which is a rare X-linked, multisystem disorder characterized by cardiomyopathy, skeletal myopathy, neutropenia, and growth retardation [53]. A lack of tafazzin results in low CL levels, and the acyl chain composition shifts toward less unsaturated species [54]. Additionally, tafazzin deficiency leads to unique developmental cardiomyopathy characterized by ventricular myocardial hypertrabeculation/noncompaction and early lethality [55]. In the hearts of adults, CL deficiency promotes the development of hypertrophic lipotoxic cardiomyopathy [56].

There is accumulating evidence on the relationship between CL remodeling and mitochondrial molecules. In cardiomyocytes with the tafazzin variant, the production of ROS from mitochondria with unhealthy CL is increased to activate Ca^2+^/calmodulin-dependent protein kinase II (CaMKII), leading to myocardial contractile disorders and arrhythmias [57]. On the other hand, in tafazzin-deficient myocardium, ROS production under hypoxic conditions is reduced and nuclear factor kappa B (NF-κB) activation is suppressed, resulting in a decrease in hypoxia-inducible factor (HIF)-1α signaling. Tafazzin-deficient murine hearts with decreased HIF-1α levels exhibit maladaptive hypertrophy with heart failure in response to pressure overload [58]. Dilated cardiomyopathy with ataxia (DCMA), hereditary cardiomyopathy caused by a mutation in a mitochondrial membrane protein, DNAJC19, presents Barth syndrome-like symptoms [59]. Mutant DNAJC19 forms a complex with prohibitin (PHB) present in the inner mitochondrial membrane, and the loss of DNAJC19/PHB complexes affects CL acylation, leading to the accumulation of CL species with altered acyl chains [60].

In addition to tafazzin, several enzymes affect the carbon chain composition of CL. Acyl-CoA lysocardiolipin acyltransferase-1 (ALCAT1; also known as LCLAT1 or LYCAT) has been identified as the enzyme responsible for the novel remodeling pathway of CL [61]. ALCAT1 is induced by oxidative stress. It exacerbates mitochondrial function [62] and is involved in mitochondrial fusion [63] and various pathological conditions, such as cardiomyopathy, Parkinson’s disease, fatty liver, and pulmonary fibrosis [64,65,66,67]. Importantly, ALCAT1 also uses phosphatidylinositol (PI) as a substrate; therefore, interpretation of its role in lipid dynamics should be carried out cautiously [68]. Acyl CoA synthetase-1 (ACSL1) is another enzyme required to incorporate LA into the CL. Deletion of ACSL1 markedly reduced C18:2 content in CL [69]. In addition, since ACSL1 expression is low in heart failure when ACSL1 is forcibly expressed to enhance the acylation of long-chain FAs, mitochondrial energy production is maintained, resulting in cardioprotection in pressure overload-induced heart failure [70].

## 4. FAs Control the Heart as Bioactive Mediators

PUFAs are released from the sn-2 position of phospholipids by phospholipase A2. Then, they are converted into a variety of unique bioactive metabolites, so-called lipid mediators, by enzymes, such as cyclooxygenase (COX), lipoxygenase (LOX), and the cytochrome p450 (CYP) family [71]. Lipid mediators are locally produced and act as signaling molecules in various physiological processes. Arachidonic acid (AA)-derived mediators are particularly active, many of which are pro-inflammatory, but some have protective functions for the heart. In addition, recent developments in mass spectrometers have made it possible to measure trace amounts of n-3 PUFA-derived metabolites, and it has become clear that these have anti-inflammatory cardioprotective functions. Furthermore, mass spectrometry has also made it possible to simultaneously measure a large number of FA metabolites from animal and human biological samples, and the dynamics of FA metabolites in various pathological conditions are being elucidated. This chapter presents the role of n-6 PUFA-derived mediators, including eicosanoids, and n-3 PUFA-derived mediators, including specialized pro-resolving mediators (SPMs), which are known to possess cardioprotective effects on heart disease.

### 4.1. n-6 PUFA-Derived Mediators

Eicosanoids, AA-derived lipid mediators such as prostaglandin (PG), leukotriene (LT), and thromboxane (TX) have various effects on cardiac function and are involved in the pathogenesis of heart diseases. Many reports have shown that eicosanoids are involved in the pathophysiology of blood vessels such as thrombus formation, endothelial function, and atherosclerosis. Eicosanoids are also known to be involved as pro-inflammatory mediators in inflammation-based pathologies for heart diseases. In systemic inflammatory conditions, the eicosanoids TXA2 and PGF2alpha act directly on the heart to cause tachycardia [72]. AA metabolites produced by cardiac 12/15-LOX are also involved in heart disease. 12/15-LOX was upregulated in heart failure of a Dahl-sensitive rat and 12-HETE, which is a major metabolite from AA by 12/15-LOX is involved in the development of heart failure by increasing monocyte chemoattractant protein 1 (MCP-1) expression [73]. In the heart of streptozotocin-induced diabetic cardiomyopathy, 12/15-LOX and inflammatory cytokines are upregulated and the disruption of 12/15-LOX reduced cardiac dysfunction and fibrosis [74].

While AA-derived mediators are pro-inflammatory and promote the development of heart disease, some mediators act protectively on the heart. Prostaglandin I2, a representative tissue-protective mediator, suppresses the onset of pressure overload-induced cardiac hypertrophy or attenuates ischemia-reperfusion injury via prostaglandin I2 (IP) receptor [75,76]. PGE2 also protects the heart from ischemia/reperfusion injury via the EP3 or EP4 receptor [77,78]. Prostaglandin D2, an AA metabolite produced by the COX pathway in cardiomyocytes protects hearts from ischemia/reperfusion injury by activating nuclear factor-erythroid 2-related factor 2 (Nrf2) [79,80]. Epoxyeicosatrienoic acids (EETs), which are metabolites of AA by CYP epoxygenases, are known to possess beneficial effects of cardiac remodeling and ischemia/reperfusion injury by various effects against inflammation, fibrosis, or apoptosis [81,82]. The metabolites of linoleic acid (LA), one of n-6 PUFAs such as AA, have been reported to affect the myocardium. 12,13-Dihydroxy-9z-octadecenoic acid (12,13-diHOME), an oxidized LA metabolite released from brown adipose tissue (BAT), is known to increase FA uptake into BAT and skeletal muscle and reduces circulating triglycerides [83,84]. Furthermore, brown adipose tissue-derived 12,13-diHOME affects cardiomyocytes directly, improves hemodynamics, and enhances cardiac function [85].

### 4.2. n-3 PUFA-Derived Mediators

Many basic studies have reported that n-3 PUFAs (which have multiple double bonds, including a third double bond from the methyl end) have cardiovascular protective effects [86]. The physiological effects of various n-3 PUFA metabolites are also important in generating cardiovascular protective effects.

18-hydroxyeicosapentaenoic acid (18-HEPE) is a primary oxidation product of EPA which exhibits anti-inflammatory and anti-fibrotic effects in the heart [87]. Transgenic mice which expressed *Caenorhabditis elegans* fat-1 protein, an n-3 desaturase that converts n-6 PUFAs to n-3 PUFAs, showed enrichment of n-3 PUFAs in almost all cells and tissues [88] and displayed resistance to numerous inflammatory diseases, including colitis, pancreatitis, osteoarthritis, atherosclerosis, obesity-linked insulin resistance, and some cancers [89]. In fat-1 transgenic mice, the decline in LV function and cardiac remodeling was suppressed even under pressure overload.

In recent years, novel n-3 PUFA-derived inflammation-regulating mediators, known as SPMs, have been identified and are attracting particular attention. SPMs are produced at the site of inflammation using n-3 PUFAs as the precursor and are actively involved in the direction of inflammation resolution [90,91,92]. Resolvin E1 and protectin D1, the representative SPMs, showed bioactivity at nanomolar levels that inhibited neutrophil migration and inflammatory cytokine production. In pathological model studies, various effects of SPMs have been reported, including the suppression of pathological angiogenesis in retinopathy [93], leukocyte infiltration, and tissue damage in ischemia-reperfusion injury in the brain and kidneys [94,95], and neutrophil infiltration in peritonitis [90]. Although the contribution of SPMs in the heart is poorly understood, SPMs are likely to reduce infarct foci in an ischemia-reperfusion model [96]. Resolvin D1 induces SPM production in the spleen, alters the phenotype of intraventricular macrophages, and inhibits fibrosis after myocardial infarction [97].

## 5. n-3 PUFAs Protect the Heart

Among the FAs, n-3 PUFAs (especially eicosapentaenoic acid [EPA] and DHA) have been validated in numerous clinical trials and used as therapeutic agents in the clinical management of cardiovascular diseases. In addition, several epidemiological studies have also revealed the beneficial effects of a high ratio of n-3/n-6 PUFA on the prevention of cardiovascular diseases [98]. Since the serum n-3/n-6 PUFA ratio is highly dependent on dietary contents, modern Western diets have approximately 15-fold higher amounts of n-6 PUFA compared to n-3 PUFA, thus increasing the risk of cardiovascular diseases. Therefore, improving the ratio of n-3/n-6 PUFA by supplementing with n-3 PUFA is expected to be one of the strategies to prevent cardiovascular diseases. In this chapter, we summarize various clinical trials using n-3 PUFAs, provide an overview of studies that elucidated the molecular mechanism of n-3 PUFAs in the pathogenesis of cardiovascular diseases, and discuss the differences between EPA and DHA.

### 5.1. n-3 PUFAs in Clinical Trials

In the 1960s, an epidemiological study showed that the Inuit people who had a diet high in n-3 PUFAs due to large consumption of fish had a significantly lower prevalence of myocardial infarction than the Danish people who mainly ate meat (other than fish) [99]. Since then, numerous interventional clinical trials have been conducted worldwide. In particular, the Gruppo Italiano per lo Studio della Sopravvivenza nell’Infarto miocardico (GISSI)-Prevenzione study, Japan EPA Lipid Intervention Study (JELIS), and Reduction of Cardiovascular Events with Icosapent Ethyl-Intervention Trial (REDUCE-IT) have accumulated evidence for the secondary prevention of ischemic heart disease [100,101,102]. Although there are a few studies that can confirm the effectiveness of n-3 PUFAs, we are yet to reach an absolute consensus because of varying results due to different study designs.

The most famous large-scale prospective study that validated the effectiveness of n-3 PUFAs in patients with heart failure was the GISSI-HF trial [103]. This trial showed that n-3 PUFAs could reduce total mortality by 9%, and cardiovascular mortality and hospitalizations were reduced by 8% in patients with symptomatic chronic heart failure who were receiving standard treatments, including aspirin, β-blockers, angiotensin-converting enzyme inhibitors/angiotensin receptor blockers, and aldosterone receptor blockers. Although the effect was small, the results were significant considering that the treatment was an addition to the standard of care. Furthermore, in patients with dilated cardiomyopathy, the addition of n-3 PUFAs to evidence-based medical therapy restored LV systolic function and functional capacity and decreased hospitalization for heart failure [104]. In addition, circulating levels of n-3 PUFAs were significantly associated with a reduced risk of heart failure with both reduced and preserved ejection fraction [105]. Although evidence is still limited, experimental reports have shown that n-3 PUFA supplementation is protective against cardiac hypertrophy and heart failure [106,107], suggesting its potential as a new treatment option for heart failure.

### 5.2. Pleiotropic Effects of n-3 PUFAs

In parallel with clinical trials, many basic research studies have been conducted to elucidate the molecular mechanism underlying the cardioprotective effects of n-3 PUFAs. Various mechanisms have been proposed for the beneficial effects of n-3 PUFAs on the cardiovascular system, including anti-arrhythmic, plasma triglyceride lowering, anti-thrombotic, anti-atherosclerotic, endothelial relaxation, blood pressure lowering, anti-inflammatory, and anti-fibrotic effects [71,86,108].

FAs are involved in a variety of bioactivities owing to their high structural plasticity. As mentioned before, while FAs are metabolized to function as bioactive mediators (see Section 4), free FAs can directly bind to receptors on the cell surface and function as ligands. In fact, orphan G-protein coupled receptors (GPCRs), such as GPR40 (Ffar1) and GPR120 (Ffar4), have been identified as receptors for long-chain free FAs [109,110]. In cardiac fibroblasts, Ffar4 is involved in the anti-fibrotic action of n-3 PUFAs [111]. Through Ffar4, n-3 PUFAs suppress transforming growth factor β (TGF-β) signaling by inhibiting the nuclear translocation of Smad2/3 and preventing pressure overload-induced cardiac fibrosis [106,112].

Free FAs also act as endogenous ligands or transcriptional regulators of various nuclear receptors and transcription factors. For example, n-3 PUFAs suppress PPAR [113,114] and the transcriptional regulator, sterol regulatory element-binding protein 1c (SREBP1c) [115], which are key molecules of lipid metabolism, thereby simultaneously controlling the transcription of downstream genes involved in triglyceride biosynthesis, including acetyl CoA carboxylase (ACC), FA synthase (FAS), and acyl-CoA synthetase (ACS). Additionally, n-3 PUFAs also affect cholesterol metabolism in the body. N-3 PUFAs enhance the activity of lipoprotein lipase which mediates the hydrolysis of triglyceride (TG) in TG-rich lipoprotein, such as chylomicron and very low-density lipoprotein (VLDL), at the luminal side of the endothelium, thereby enhancing the catabolism of VLDL to lower the blood VLDL level [116]. Furthermore, n-3 PUFAs suppress the generation of small dense LDL by reducing the expression of cholesterol ester transfer protein (CETP) which shuttles cholesteryl ester from HDL to apo-B protein [117]. Thus, n-3 PUFAs have various improving effects on lipid profile, and as a result, exert a strong anti-arteriosclerotic effect in combination [118,119].

Furthermore, n-3 PUFAs can be incorporated into the phospholipid bilayer of cell membranes and can affect membrane fluidity, lipid microdomain formation, and signaling across membranes. Cardiomyocytes enriched with n-3 PUFA (DHA) in the cell membrane showed the alteration of cholesterol homeostasis that increased cholesterol biosynthesis, cholesterol efflux, and free cholesterol pool size, compared to n-6 PUFA (AA)-enriched cardiomyocytes [120]. Additionally, n-3 PUFAs modulate ion channels, such as L-type calcium channels and sodium channels within the cell membrane of cardiomyocytes, resulting in an anti-arrhythmic effect [121,122].

### 5.3. The Differences between EPA and DHA

To the best of our knowledge, there are no interventional studies comparing the preventive and therapeutic effects of DHA or EPA alone on cardiovascular diseases; therefore, it is difficult to conclude which FA contributes to the cardioprotective roles of n-3 PUFAs. A recent REDUCE-IT study [102] (high-dose, high-purity EPA intake 4 g/day) showed positive results supporting the potential of n-3 PUFAs, while the Vitamin D and Omega-3 Trial (VITAL) and A Study of Cardiovascular Events in Diabetes (ASCEND) studies [123,124] published at the same time (normal dose of EPA and DHA intake of 1 g/day) showed negative results for the effectiveness of n-3 PUFAs. These results suggest that the use of high doses of high-purity EPA is necessary to exert cardioprotective effects. However, the results of the most recently published Statin Residual Risk Reduction With Epanova in High CV Risk Patients With Hypertriglyceridemia (STRENGTH) study (EPA [4 g] and DHA) were negative, rekindling the debate on the efficacy of n-3 PUFAs. On the other hand, in the Omega-3 Acid Ethyl Esters on Left Ventricular Remodeling After Acute Myocardial Infarction (OMEGA-REMODELED) study, the patients with “post-acute myocardial infarction” who were treated with high-dose EPA and DHA showed a reduction in adverse LV remodeling and non-infarct myocardial fibrosis [125]. High-dose, high-purity EPA administration may be the most effective requirement, but this is not yet conclusive. Therefore, it is important to select the appropriate patient population for treatment with n-3 PUFAs based on the patient background, intervention, and outcomes of each clinical trial.

In basic research, there is still a lack of evidence to determine the differences between the roles of EPA and DHA in cardiovascular diseases, especially heart failure. Several studies have reported anti-oxidant effects of EPA not found in DHA, suggesting EPA-specific vascular benefits [71]. Interestingly, DHA is more abundant than EPA in the heart, but the reason and significance for this observation are unknown. Of note, the mechanism by which DHA is selectively taken up in the brain and retina has been reported [126,127,128], but is not well understood in the heart.

## 6. Conclusions

As discussed above, FAs play three important roles in living organisms: (1) energy sources, (2) components of membrane phospholipids, and (3) bioactive mediators. The dynamics of FAs in the heart are elaborate and complex. The conventional theory that ATP production in the healthy heart is dependent on FAO but shifts to dependence on glucose metabolism in pathological conditions, such as ischemia and heart failure, has become more complicated under the modern lifestyle exposed to metabolic stress. The development of lipid measurement technology enables a highly accurate qualitative assessment of FAs and their derivatives, and the roles of various molecules that regulate FA dynamics are becoming clear. Understanding and accumulating knowledge of the complex dynamics of FAs at the cellular, tissue, and systemic levels are expected to establish new therapeutic targets for heart failure.
